# Transcriptomic Evidence Reveals Low Gelatinous Layer Biosynthesis in *Neolamarckia cadamba* after Gravistimulation

**DOI:** 10.3390/ijms24010268

**Published:** 2022-12-23

**Authors:** Mirza Faisal Qaseem, Kaili Wang, Haoqiang Yang, Shuai Zhao, Huiling Li, Ai-Min Wu

**Affiliations:** 1 State Key Laboratory for Conservation and Utilization of Subtropical Agro-Bioresources, Guangzhou 510642, China; 2 Guangdong Key Laboratory for Innovative Development and Utilization of Forest Plant Germplasm, College of Forestry and Landscape Architectures, South China Agricultural University, Guangzhou 510642, China; 3 Department of Environmental Science and Forestry, Connecticut Agricultural Experiment Station, 123 Huntington Street, New Haven, CT 06511, USA; 4 State Key Laboratory for Conservation and Utilization of Subtropical Agro-Bioresources, Guangxi Research Center for Microbial and Enzyme Engineering Technology, College of Life Science and Technology, Guangxi University, Nanning 530004, China; 5 Guangdong Laboratory of Lingnan Modern Agriculture, Guangzhou 510642, China

**Keywords:** bending stress, G layer, *Neolamarckia cadamba*, transcriptome, plant hormones

## Abstract

Trees can control their shape and resist gravity by producing tension wood (TW), which is a special wood that results from trees being put under stress. TW is characterized by the presence of a gelatinous layer (G layer) and the differential distribution of cell wall polymers. In this study, we investigated whether or not gravistimulation in *N*. *cadamba* resulted in TW with an obvious G layer. The results revealed an absence of an obvious G layer in samples of the upper side of a leaning stem (UW), as well as an accumulation of cellulose and a decrease in lignin content. A negligible change in the content of these polymers was recorded and compared to untreated plant (NW) samples, revealing the presence of a G layer either in much lower concentrations or in a lignified form. A transcriptomic investigation demonstrated a higher expression of cell wall esterase- and hydrolase-related genes in the UW, suggesting an accumulation of noncellulosic sugars in the UW, similar to the spectroscopy results. Furthermore, several G-layer-specific genes were also downregulated, including fasciclin-like arabinogalactan proteins (*FLA*), beta-galactosidase (*BGAL*) and chitinase-like proteins (*CTL*). The gene coexpression network revealed a strong correlation between cell-wall-synthesis-related genes and G-layer-synthesis-specific genes, suggesting their probable antagonistic role during G layer formation. In brief, the G layer in *N*. *cadamba* was either synthesized in a very low amount or was lignified during an early stage of growth; further experimental validation is required to understand the exact mechanism and stage of G layer formation in *N*. *cadamba* during gravistimulation.

## 1. Introduction

Wood is an outstanding material for the timber and paper industries due to its excellent mechanical properties and high lignocellulosic content, and as technologies emerge, wood qualities are constantly being improved, including the development of popular lignin-free wood for biofuel production [1,2]. Wood formation involves a variety of complex events, such as cambium division, secondary wall deposition and program cell death, to form a fully differentiated secondary vascular bundle [3]. However, when a plant is subjected to mechanical stress, a particular type of wood known as reaction wood is formed. There are two types of reaction wood: tension wood (TW) and compression wood (CW). Tension wood forms on the upper side of the stem and CW forms on the lower side of the stem in hardwoods, whereas the opposite is true for softwoods [4,5,6,7]. Both TW and CW differ in their anatomy, physiology, chemical and machinal properties. TW in some arboreal eudicots forms an unusual wood fiber know as gelatinous fiber, or G fiber, which can form a gelatinous layer (G layer) as the innermost layer of a multilayered cell wall, aiding in fiber contraction during maturity and functions in altering plant axes and the shape [8,9,10]. The G layer has been found in approximately half of dicotyledonous species, and has also been reported in thorns, coiling tendrils, contractile roots, peduncles and the phloem [2,11]. It is possible that G fibers of various origins might have a unique and shared role in the bending of axes by producing high tensile stress [12].

The G layer’s development appears to begin immediately after plant stems are bent, resulting in an increased cell thickness and thinner S1/S2 cell wall layers prior to the G layer’s deposition [13]. Furthermore, some studies suggest that the G layer can partially or completely replace the S2 layer, whereas some others suggest that it is a complete replacement of the S3 layer [11,13]. The types of G layers that form in angiosperms vary greatly; for example, a multilayered G layer has been seen in *Laetia procera* [14,15], whereas *Simarouba amara*, which was previously known for having TW without a G layer [16], was recently discovered to have a G layer in TW during the early stages of cell wall maturation, which later lignifies and disappears [15]. Thus, many plant species previously thought to lack a G layer may have a G layer that lignifies prior to investigation [17]. Biochemically, the G layer is characterized by the presence of a low lignin [18] and high cellulose content, although some plants may also have non-cellulosic polysaccharides, such as xyloglucans [8,9,10]. Other components of the G layer are not yet known, although some previous research has shown it to originate from proteins and mineral ions [19], as the G layer in the *Populus* consisted of 72 proteins; however, some of these were also involved in lignin biosynthesis, while others were cytoskeleton proteins [20]. The G layer in TW induces specific properties, such as an increased microporosity by changing the size and shape of mesopores, which are responsible for the G layer matrix’s expansion during maturity [21]. Cellulose in the G layer is highly crystalline, since G layer microfibrils form crystalline macroaggregates [20,22], and the orientation of cellulose microfibrils differs from that of normal cells, as they are oriented parallel to the axis of the fibers and are responsible for tensile strength generation [23,24].

At a molecular level, various genes and transcriptional factors have been shown to be involved in the regulation of G layer formation. The *BGALs* are the enzymes responsible for the modification of *RG-I* pectin in the G layer in flax *LuBGAL1* and/or *LuBGAL2*, hydrolyzing high-molecular-weight galactans and converting them into a galactan-rich layer (Gn layer), which is then converted into a G layer [25,26]. Flax *bgal1* and *bgal2* mutants have higher levels of cell-wall-associated galactans and lower levels of crystallin cellulose, as well as a reduced Gn layer to G layer convergence ability and stem strength [25]. The association between the negatively charged galacturonic acid of pectin and its transporters, such as arabinogalactan proteins (*AGPs*) regarded as *AGPs* with an *RG*–I interaction, may be responsible for mesoporosity during G layer formation [21]. This association alters the pectin alignment and decreases its crosslinking in the cell wall, resulting in increased porosity in the pectin network [27]. Arabinogalactan type II proteins isolated from *Populus* TW were discovered to be conserved between its cellulose fibrils and were responsible for gel structure formation [9]. Fasciclin-like arabinogalactan proteins (*FLAs*) are claimed to be important in the regulation of TW formation, secondary cell wall growth and mechanical properties of stem cells, and in the maintenance of the axial orientation of cellulose microfibrils in the G layer [28,29]. Double mutations of *FLA11* and *FLA12* increase the lignin content and microfibril angle, whilst also reducing the polysaccharide content, stem tensile strength and stiffness [30]. Likewise, *FLA5* overexpression in cotton fibers significantly increases stem strength and also affects cellulose synthesis and microfibril angles [31]. TW-specific *FLA6* protein alteration in poplar not only reduced stem strength and stiffness and lignin and crystalline cellulose content but also altered the expression of nine other *FLA* genes involved in lignin and cellulose synthesis [32]. The *CTLs* are also abundant in both TW as well as CW [33]. Xylem-specific *AtCTL2* and its close homologue *AtCTL1* are involved in the synthesis of the cell wall in different plants [34,35]. Both of these genes, i.e., *AtCTL2* and *AtCTL1,* are co-expressed with *CESA* genes in the primary and secondary wall, respectively. It was proposed that they contribute to cellulose crystallinity and cellulose microfibril assembly, with a secondary effect on microfibrils and cross-links with hemicelluloses [35]. A group of highly expressed chitinase-like genes in phloemic G-fibers of flax was shown to be divergent from all other chitinase groups [36]. However, members of this group are non-homologous to *AtCTL1* and *AtCTL2*.

*N*. *cadamba* is a tropical, evergreen, and fast-growing tree native to south China, southeast Asia and the Pacific; however, due to its rapid growth, it has been introduced in Africa and South America in areas with suitable climatic conditions for its growth [37]. Several previous studies, as well as a recent one, have shown that when grown in identical environmental conditions, *N*. *cadamba* can grow at a similar rate to, if not outperform, widely cultivated fast-growing trees such as *Populus* and *Eucalyptus*, etc. [38]. Thus, *N*. *cadamba* wood can be used as a low-cost, abundant feedstock for bioenergy production [39,40]. TW is the ideal bioresource to produce biofuels due to its high cellulose content. Additionally, the G layer of TW is unlignified and porous, which reduces the recalcitrance of the biomass and makes it simple to degrade without the need for abrasive or expensive pretreatments [41,42]. In addition, there have been no comprehensive studies on the mechanism of TW and G layer formation in *N. cadamba*. Thus, the current project aimed to investigate the comprehensive anatomical changes and transcriptome reprogramming during G layer formation in *N. cadamba* by imposing artificial bending stress.

## 2. Results

### 2.1. Microscopic Analysis

**Figure 1:**
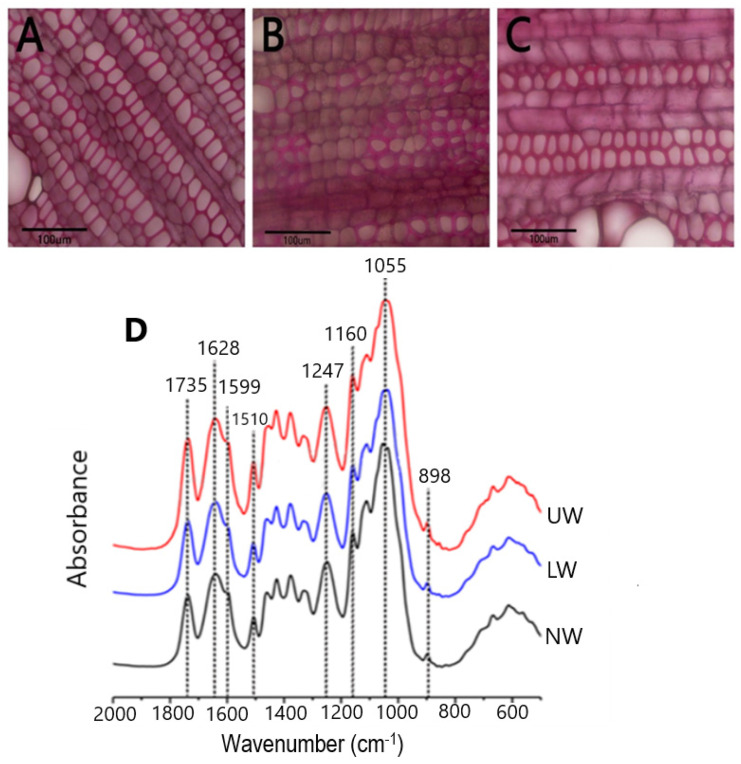
Microscopic observation of stem sections. (**A**) Normal wood (NW), (**B**) lower wood (LW) and (**C**) upper wood (UW) formed in response to bending stress. Staining was performed using phloroglucinol. No clear G layer formation was seen in TW. (**D**) Results from FT-IR spectroscopy of *N. cadamba* wood: infrared spectra of NW, LW and UW samples.

In plants, TW develops in response to bending stress, and TW with an cellulose abundant G layer on the lumen side of fibers is considered to be the most commonly TW observed in many species [11,12]. Young *N. cadamba* plants were placed horizontally for about thirty days (120-day-old plantlets were allowed to bend until 150 days old) to study G layer formation during gravistimulation. To visualize the G layer, samples were stained with phloroglucinol, and the results showed that UW samples were stained red and, therefore, *N. cadamba* does not have an obvious G layer (Figure 1A–C).

### 2.2. Infrared Spectrum Analysis

To confirm the microscopic findings, we used Fourier transform infrared (FT-IR) spectroscopy to look for differences in chemical bonding and structure between the different cell wall polymers in all samples. The relative height of the peaks was used to calculate the content of the cell wall components. Literature searches [43,44] revealed that the peak at 1735 cm^−1^ in the spectrum represents the acetyl group, while the peak at 1247 cm^−1^ corresponds to the C-O linkage in the acetyl group. The peaks at 1628 cm^−1^ and 1599 cm^−1^ indicate the presence of C-Ph bond and C=C bonds in lignin, respectively (Figure 1D). Another peak at 1510 cm^−1^ corresponds to the C=C bond in the aromatic ring of lignin. The characteristic absorption peak of xylan was detected at 1055 cm^−1^, while the peak at 1160 cm^−1^ showed arabinosyl substitution on the xylan backbone. The β-glycosidic bonding between the sugar units is represented by the peak at 898 cm^−1^. The presence of peaks for acetyl and arabinoxylan confirms the presence of acetylated arabinoxylan type of hemicellulose in *N. cadamba*. By comparing the lignin peaks at 1599 cm^−1^ and 1510 cm^−1^, it is evident that the lignin content decreases from LW to NW, while there is no significant difference in lignin content between the UW and NW samples. The cellulose content in LW and UW was similar, but slightly higher than that in NW. In addition, the hemicellulose content was higher in LW and UW than in NW (Table S1).

### 2.3. Transcriptome Profiling Reveals Changes in Gene Expression in Normal and Reaction Wood Samples

**Figure 2:**
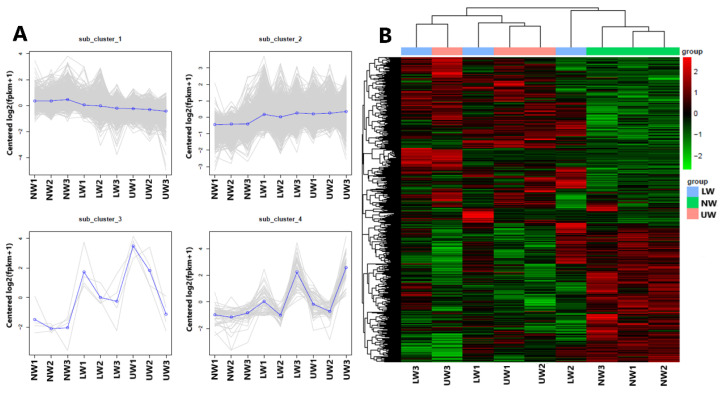
Results from multivariate analysis. (**A**) The first four principal components and distribution of genes in these clusters, and (**B**) clustering heat map of differentially expressed genes in different wood samples.

To further check the transcriptome profile differences between NW, LW and UW samples, three young stems from each group were sampled for RNA-Seq analysis. After quality evaluation, 416,038,776 clean reads were generated (Table S2), which were genomically aligned at over 93%, with 94.07–95.29% of the matched sequence reads uniquely aligned with the reference genome sequence (Table S3). The high repeatability of data for all three biological replicates was confirmed via high Pearson correlation coefficient (r^2^ > 0.90) values (Figure S1). All differentially expressed genes (DEGs) were used for cluster analysis, and the whole data were divided into three clusters (Figure 2A). Based on the DEGs data, the three NW1-3 and UW1-3 samples were grouped into two different clusters, while the LW1-3 samples were worse and were grouped into NW and UW groups. Moreover, the gene expression patterns of NW, LW and UW samples were significantly different. It is clear from the heat map (Figure 2B) that genes with higher expression in NW samples have a lower expression in LW and UW samples, and vice versa.

### 2.4. Differential Expression of Genes (DEGs)

**Figure 3:**
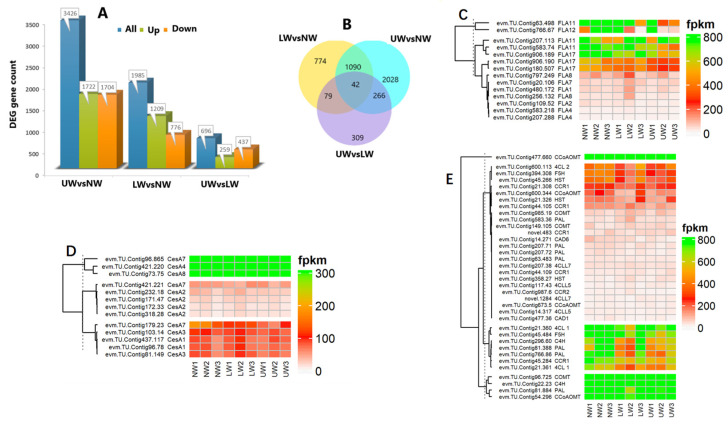
Differential expression of significant genes. (**A**) Number of DEGs up- or downregulated in the studied wood samples. (**B**) Venn diagram showing shared and unique unigenes among different studied samples. (**C**) Differential expression of FLA genes. (**D**) Expression of cellulose synthesis-related genes. (**E**) Differential expression of genes responsible for lignin biosynthesis.

In the present study, genes that were significant at log2FC ≥ 1 or *p* value ≤ 0.05 were regarded as DEGs and were selected to understand the degree of alteration in gene expression and key genes associated with G layer formation and bending stress tolerance in *N. cadamba*. In total, 6107 DEGs were identified across all samples, including 1985 for LW vs. NW, 696 for UW vs. LW and 3426 for UW vs. NW. In the LW vs. NW group, 1209 genes were upregulated and 776 genes were downregulated; in the UW vs. LW group, 259 genes were upregulated and 437 genes were downregulated; and in the UW vs. NW samples, 1722 genes were upregulated and 1704 genes were downregulated (Figure 3A). In total, 42 DEGs were expressed in all samples, while 266 were common between UW vs. LW and UW vs. NW, 79 were common between UW vs. LW and LW vs. NW, and 1090 were common between UW vs. NW and LW vs. NW (Figure 3B).

### 2.5. Functional Characterization of DEGs

GO term annotation classification was used to describe the functions of significant DEGs. In the LW vs. NW samples, 39 significant GO terms were recorded from DEGs, of which 14 genes were upregulated and GO terms were categorized as cellular components (CF) and molecular functions (MF). In the CF category, genes are associated with cell walls, extracellular regions, and coverage or encapsulation of extra cellular regions. Genes in the MF category include those that “bind to heme and tetrapyrrole groups”, “transfer hydroxyl groups”, and are involved in “xyloglucosyl transferase” and “phosphotransferase activities” (Figure S2A,B).

Next, we recorded twenty significant GO terms for DEGs in the UW vs. LW groups. Among them, GTPase, glucosyltransferase, pyrophosphatase, transferase and hydrolase activities were associated with upregulated DEGs. Among the upregulated genes in the UW vs. LW group, a significant GO term for the MF category “transcription regulator activity” was found (Figure S3A,B).

Finally, more informative GO terms were recorded for UW vs. NW, containing 41 upregulated genes and 29 downregulated genes. Upregulated genes contain MF GO terms for activities such as phosphorelay sensor kinase, protein histidine kinase, and phosphotransferase. In addition, they act as transmembrane transporters for sulfates and other sulfur-containing compounds, glycosyl groups, anions, inorganic anions, etc., with deaminase, ATPase, ligase and antioxidant activities. The GO terms of cellular components are associated with cell wall and extracellular structures. Only nine “biological processes” terms were found in the upregulated genes of UW vs. NW, namely “transport of ions and sulfur compounds”, “glutamine family amino acid biosynthetic process”, “glutamine metabolic process” and “response to oxidative stress” (Figure S4A,B).

### 2.6. KEGG Pathway Enrichment Analysis

KEGG pathway enrichment analysis revealed that the “plant hormone signal transduction” pathway was the only pathway significantly enriched for LW vs. NW upregulated genes (Figure S5a), whereas no pathway was significantly enriched for UW vs. LW upregulated genes, but the “starch and sucrose metabolism” and “plant hormone signal transduction” pathways had the highest number of genes (Figure S5b). Likewise, for the UW vs. LW downregulated genes, no pathway was significantly enriched, but the “carbon metabolism” pathway had highest number of genes. The downregulated genes in the UW vs. NW samples were significantly enriched for pathways involved in the “phagosome” and many other sugar metabolism pathways. The pathways involved in “zeatin biosynthesis” and “plant hormone signal transduction” were significantly enriched with upregulated genes in UW vs. NW (Figure S5c).

### 2.7. Differential Expression of Genes Involved in Different Biological Processes

Based on the results of the functional enrichment analysis, genes related to cell wall synthesis, hormonal signaling and sugar metabolism as well as some gravity and G layer-related genes are discussed in detail. The differential expression patterns of these important genes will be discussed in detail in the subsequent sections.

#### 2.7.1. Differential Expression of Genes Related to Carbon Partitioning and G Layer Formation

Key enzymes for UDP–glucose conversion, i.e., *UDP-xylose synthase* (*UXS*) *UXS1*, *UXS6*, *UDP-glucuronic acid decarboxylase4* (*UGD4*), and *UDP-glucose 4-epimerase1* (*UGE1*), were more abundant in UW than in NW (Table S4). Similarly, uridine monophosphate kinase (*evm.TU.Contig906.3*), which synthesizes UDP, was upregulated in UW. Other general sugar-activating enzymes such as malate synthase, isocitrate dehydrogenase (*evm.TU.Contig66.167*), *lactate dehydrogenase* (*evm.TU.Contig294.378*), raffinose synthase (*evm.TU.Contig102.2*), and others were also upregulated in UW samples (Table S4), indicating an increased flux of sugar towards the upper side of the stem. In addition to sugar synthesis-responsive genes, many genes encoding sugar transporters, RNA-binding proteins, potassium and other ion (Ca, S, Fe, etc.) channels, H^+^-transporting ATPase, and amino acid carriers showed increased expression in UW (Table S4), providing molecular evidence for more sugar and ion accumulation in UW samples. Many *FLA* genes, such as *FLA2* (*evm.TU.Contig109.52*), *FLA8* (*evm.TU.Contig256.132*), *FLA11* (*evm.TU.Contig583.74* and *evm.TU.Contig63.498*), *FLA12* (*evm.TU.Contig766.67*), and *FLA17* (*evm.TU.Contig906.189, evm.TU.Contig180.507* and *evm.TU.Contig906.190*) were downregulated in the UW samples (Figure 3C), thus providing evidence of a low or lignified G layer in *N. cadamba*.

#### 2.7.2. Expression of Genes Related to Cell Wall Polymer Synthesis

The plant cell wall is composed of cellulose, hemicellulose and lignin, and any alteration in external stimuli affects the organization of these cellular components, which indicates the role of cell wall composition and signaling in stress tolerance. In the present study, the *cellulose synthesis genes (CesA*) *CesA4*, *CesA8* and *CesA7* had the highest expression in all samples (Figure 3D). Comparatively, CesA7 encoding the unigene *evm.TU.Contig96.865* was highly expressed in NW samples and downregulated in UW samples. The *evm.TU.Contig171.47* and *evm.TU.Contig232.18* associated with *CesA2* were upregulated in NW and downregulated in UW. Likewise, *evm.TU.Contig81.149* and evm.TU.Contig103.14 associated with *CesA3* were also highly expressed in NW compared to UW, indicating greater accumulation of cellulosic sugars in NW compared to UW. Similarly, genes related to hemicellulose biosynthesis, such as the *irregular xylem* (*IRX*) *IRX9, IRX10L, IRX14, IRX7*, and *trichome birefringence-like proteins* (*TBLs*), were highly expressed in NW compared to UW and LW. The *cellulose synthase-like A 9* (*CslA9*) gene, involved in mannan biosynthesis, was highly expressed in NW than in UW. *Cellulose synthase-like G1* (*CslG1*) was upregulated in UW and LW and downregulated in NW samples.

Many genes involved in the phenylpropanoid pathway were upregulated in all samples; for example, *evm.TU.Contig477.660* associated with *caffeoyl-CoA O-methyltransferase* (*CCoAOMT*) was highly expressed in all samples. Other lignin-synthesizing genes, classified according to their expression into three clusters *evm.TU.Contig96.725* (*caffeic acid O-methyltransferase; COMT*), *evm.TU.Contig22.23* (*cinnamate 4-hydroxylase, C4H*) and *evm.TU.Contig81.884* (*phenylalanine ammonia-lyase*, *PAL*), were highly expressed in all samples; however, their expression was higher in NW than in UW, confirming the FTIR results, with little variation in content between cellulose and lignin. The minor changes in cell wall polymers, especially in the content of cellulose and lignin, suggest that the G layer is either synthesized but in small amounts, or lignified owing to the maturity of the studied plants. A variety of functioning laccase enzymes involved in both lignin biosynthesis and degradation were found to be inconsistently expressed in UW and LW samples. *Laccase* (*LAC2*) and *LAC17* were upregulated in LW and UW samples, while *LAC10*, *LAC17* and *LAC22* were downregulated in UW samples (Figure 3E).

**Figure 4:**
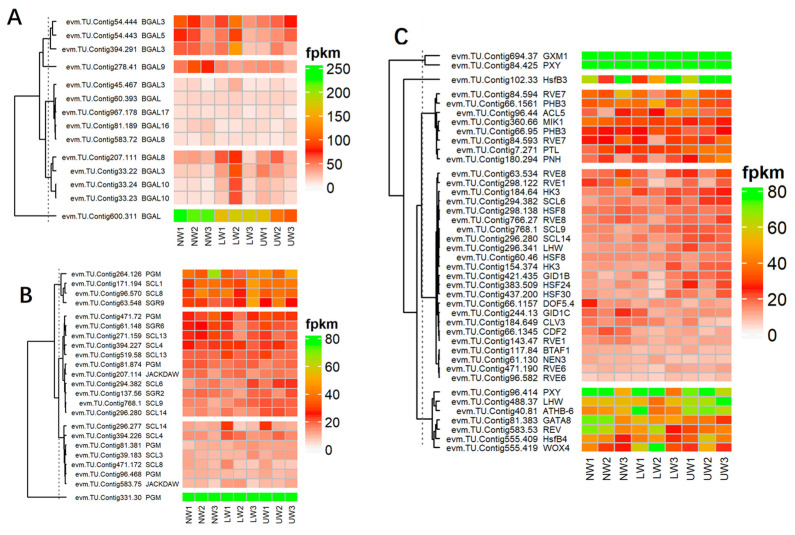
(**A**) DEG of β-galactosidase-encoding genes involved in G layer formation. (**B**) Differential expression of gravity-related genes. (**C**) Differential expression of genes and transcriptional factors associated with vascular bundle and cambium development and proliferation.

Many cell-wall esterase and signaling molecule-related genes were altered—for example, the expression of xyloglucan *endotransglucosylase/hydrolase* (*XTH*) genes, namely *XTH9*, *XTH33*, *XTH10* and *XTH27*, was upregulated in NW and decreased in UW and LW (Table S5). Similarly, many members of *β-glucosidase* (*BGLU*) genes and *cellulose catabolism genes* (*CELs*) were differentially expressed in UW and NW samples. The expression of *BGLU24*, *BGLU13* and *BGLU24* was upregulated in UW samples, while *CEL1*, *CEL3* and *CEL5* were significantly downregulated. In contrast, *BGAL* genes were highly expressed in NW compared to LW and UW. *BGAL5* and *BGAL3* were highly expressed in NW, while their expression was gradually decreased in LW and UW (Figure 4A).

Cell-wall-signaling-related genes, including *annexin* (*evm.TU.Contig12.235* and *evm.TU.Contig280.115*) and *IQ-Domain14* (*evm.TU.Contig279.14* in UW and *evm.TU.Contig539.5* in LW) were downregulated, while *IQD1* (*evm.TU.Contig154.187*), *IQ*-*Domain31* (*evm.TU.Contig471.101*) and *IQ-Domain32* (*evm.TU.Contig555.180*) were upregulated in both UW and LW samples. The expression of vascular-related *NAC*-domain protein 4 (*VND4*) was downregulated in both UW and LW samples, whereas the expression of *VND7* was highly upregulated in UW samples. Similarly, secondary-wall-associated *NAC* domain protein 2 (*SND2*), a *NAC* transcriptional factor (TF) involved in secondary cell wall fiber development, was downregulated in UW vs. NW samples. In the same way, many *NAC* and *myeloblastosis viral oncogene homolog* (*MYB*) TFs were also significantly expressed between UW and LW samples.

#### 2.7.3. Expression of Genes Related to Epigenetic Regulation and Gravity

In this study, the expression of the *evm.TU.Contig331.30* unigene was higher in LW than in UW and NW, while all other unigenes associated with phosphoglucomutase (PGM) were more highly expressed in NW and UW than in LW. The expression of *evm.TU.Contig207.114* and *evm.TU.Contig583.75* associated with *JACKDAW*, which is part of the cluster that regulates the *SHORT-ROOT* gene and maintains the radial patterning and stem cells, was upregulated in LW. *Shoot gravitropism* (*SGR*) is also involved in the gravity response, and these mutations result in a complete loss of the gravity response. In the present study, the expression values of *SGR2, SGR6* and *SGR9* did not differ much in all studied samples. Actin organization and orientation are also important for gravity sensing and response in plant organs—for instance, *actin response proteins* (*ARP*) regulate actin organization and the formation of actin bundles around the amyloplasts. In the present study, *ARP* 2, 3, 5, 7, and 9 genes were upregulated in NW and downregulated in UW, whereas *ARP4* and 6 were upregulated in UW (Figure 4B).

#### 2.7.4. Alteration in Xylem- and Vascular Cambium-Related Gene Expression during Leaning Stress

The main characteristics of TW development are asymmetric cambial growth, reduced size and number of vessels, and disrupted phloem structure. In our study, many genes encoding TFs from the *homeodomain leucine zipper* (*HDZip*) family were differentially expressed, but these members are involved in stress tolerance rather than TW or G layer development. For example, *homeobox-leucine zipper protein* (*ATHB14*) was highly expressed in all samples and was more highly expressed in NW. Likewise, the expression of *ATHB15* (*evm.TU.Contig46.13*) was found to be higher in NW compared to UW and LW, and a similar expression pattern was observed in other *ATHB* members. Alterations in the expression of many genes and transcriptional factors responsive to phloem and cambium development were recorded in UW and LW samples. Other TFs responsible for phloem development, such as *DNA-binding with one finger* (*Dof*) and no apical meristem (*NAM*), were also differentially expressed. In the present study, Dof genes previously described in *Populus* [45], which are associated with cambial development, were differentially expressed. In addition, two members, *evm.TU.Contig66.1345* (*CDF2*) and *evm.TU.Contig66.1157* (*DOF5*.4), were downregulated in the UW samples (Figure 4C). Many studies have demonstrated the association of *Dof* family TFs with phloem development [46,47]. Thus, our results confirm the involvement of *MYB*, *Dof* and *NAM* TFs in cambium and phloem development during mechanical bending in *N. cadamba*. Similarly, many genes regulating cambial development and activity were also differentially expressed. Evidence suggests that, like in apical meristems, *WUSCHEL*-*CLAVATA*-like regulator model also regulates cambial meristems. *WOX4* is a *WUSCHEL*-related *HOMEOBOX* gene that promotes procambial/cambial cell proliferation by exerting influence on the *TDIF/CLE41/CLE44-TDR/PXY* signaling pathway [48]. *WOX4* and *WOX8* were upregulated in UW and LW and showed weak expression in NW samples. In the present study, *CLV3*, a putative ortholog of *CLAVATA3*, was downregulated in UW and highly expressed in NW and LW, respectively (Figure 4C). The expression of *PINHEAD* was higher in UW and the *ANT* homolog of *AINTEGUMENTA* was upregulated in UW, indicating their role in modulating cambial cell division and proliferation [49]. *PINHEAD* gene overexpression regulates the initiation [50] and maintenance [51] of the vascular cambium. The vascular root patterning in *Arabidopsis* roots is regulated by *SHORTROOT* (*SHR*) and *SCARECROW* (*SCR*), which belong to the *GRAS* TFs family [52,53]. In *N. cadamba*, only one homolog of *SHR* (*evm.TU.Contig66.704)* was detected in the opposite wood and was downregulated, while six SCR-like samples (*SCL14*, *SCL6* and *SCL9*) were upregulated in UW samples (Figure 4C).

#### 2.7.5. Alteration in the Expression of Hormones Synthesis and Signaling Responsive Genes

**Figure 5:**
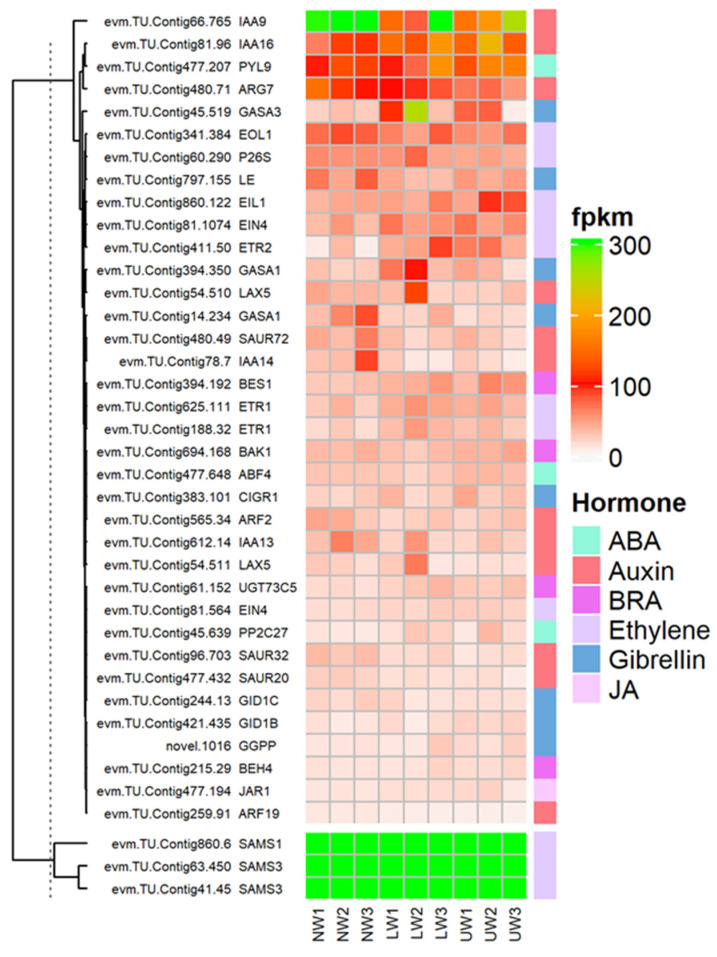
Differential expression of genes and TFs associated with hormonal signaling and synthesis-related genes in the studied samples.

Plant hormones, as core regulators of plant growth and development, not only regulate internal developmental processes, but also help plants to survive harsh external environments [54,55]. Regarding brassinosteroid (BR), *BRI1-ASSOCIATED RECEPTOR KINASE 1* (*BAK1*)-associated unigenes *evm.TU.Contig694.168* and *evm.TU.Contig16.193* showed higher expression in UW samples, while *evm.TU.Contig256.105* was highly expressed in both LW and NW (Figure 5). These two factors are associated with BR perception and extreme twist and form an integral BR-binding pocket. The expression of *BRASSINOSTEROID-INSENSITIVE 4* (*BIN4*), a master negative regulator of the BR signaling pathway, was upregulated in LW and UW samples (Figure 5). Similarly, the expression of *BRASSINOSTEROID ENHANCED EXPRESSION 1* (*BEE1*), which is involved in the early response required for brassinosteroid (BR) action, was upregulated in NW and LW compared to UW samples (Figure 5). The expression of *BES1/BZR1* homolog protein 4 (*BEH4*) (*evm.TU.Contig215.29* and *evm.TU.Contig434.57* [47]) was upregulated in UW and LW samples, while *BES1/BZR1* homolog protein 2 (*BES1*) (evm.TU.Contig394.192) was upregulated in UW (Figure 5). *PROTEIN PHOSPHATASE 2A* (*PP2A*), an activator of *BZR1* and *BES1*, was downregulated in UW and LW (Figure 5).

Ethylene and its precursor synthesis-related genes, *S′adenosyl-l-methionine* (*SAMs*), were upregulated in all three wood samples. The expression of *1-aminocyclopropane-1-carboxylic acid* (*ACC*), an immediate precursor of ethylene biosynthesis, was upregulated in LW and downregulated in UW and NW (Figure 5). The expression of ethylene receptors (*ETR1*) (*evm.TU.Contig625.111; evm.TU.Contig188.32*) was higher in LW, while *ETR2* (*evm.TU.Contig411.50*) was highly expressed in UW samples. *Ethylene-overproduction protein 1* (*ETO1*), an essential stabilizer of *ACC* synthase (*ACS*) enzyme during ethylene biosynthesis, was more highly expressed in LW than in UW and NW.

**Figure 6:**
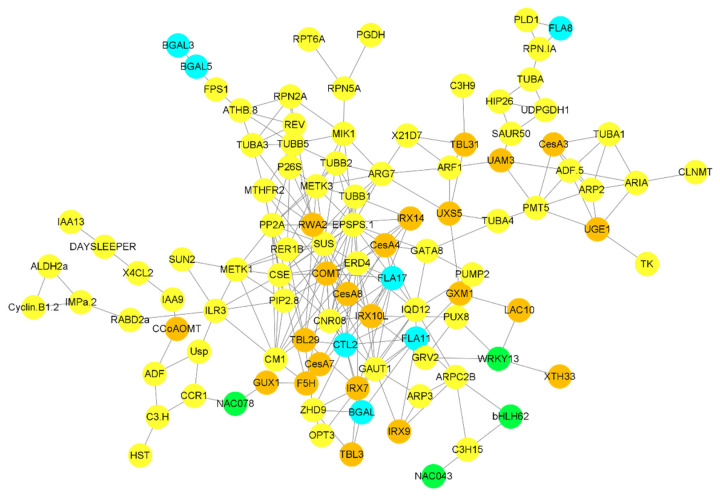
Correlation network between G layer-forming genes and other genes and TFs involved in different biological processes. Only highly expressed and correlated genes were used to build the network. A sky-blue color indicates genes involved in G layer formation, brown circles highlight genes involved in cell wall biosynthesis, green circles highlight TFs, and all other genes highly correlated with G layer forming genes are highlighted in yellow.

The expression of the ethylene response factors (*ERF*) *ERF003, ERF010, ERF024, ERF3* and *ERF3* associated with the *APETALA2*/ethylene responsive factor (*AP2*/*ERF*) TF was highest in the UW samples (Figure 5). The expression of *XRN4*, an integral component of ethylene signaling, was the same in all samples (Figure 6). Again, *CTR1*, a negative regulator of ethylene signaling, had a similar expression in NW and UW and slightly lower expression in LW. The expression of *EIN2* was similar in both NW and UW and slightly lower in LW. The expression of *EIL1* was higher in UW than in NW and LW. *EIN3*-binding *F-box protein 2* (*EBF2*), which stabilizes the synthesis of *EIN3* and *EIL1* proteins, was more highly expressed in NW and LW than in UW.

Many vital plant processes related to plant growth and development are regulated by auxins, either through their individual influence or by regulating the expression of other plant hormones. In the present study, auxin responsive unigenes were associated with ten ARF genes, among which *ARF10* was highly expressed in UW, followed by *ARF1,* which was highly expressed in LW. Six members of the *Aux*/*IAA* gene family, namely *IAA8*, *IAA9*, *IAA13*, *IAA14*, *IAA16* and *IAA27*, were differentially expressed in all samples. The expression of *AUX22B*, *AUX22D* and *AUX28* was reduced in UW and increased in LW. In addition to genes responsible for auxin synthesis, many other genes involved in making conjugates of auxin with other hormones or molecules showed higher expression in UW—for example, *GH3.17* was highly expressed in UW samples (Figure 5). Many auxin transporters were differentially expressed in UW, LW and NW samples, e.g., *LAX5* had similar expression in all samples, and *ABCB19*, *MES17*, *MES10* and *MES11* were highly expressed in UW, while *PIN1a* was upregulated in UW and LW samples (Figure 5).

Abscisic acid, also known as stress hormone, is a plant hormone involved in the modulation of many biological and cellular processes as well as stress tolerance. In the present study, the expression of ABA transporter protein *ABCC2* (*evm.TU.Contig1.103*) was high in both UW and NW. The expression of *PYL4* and *PYL9* nucleocytoplasmic receptors was upregulated in UW, while the expression of *PP2C40* (*evm.TU.Contig16.680*) was downregulated in UW samples, probably due to the binding of ABA to nucleocytoplasmic receptors (Figure 5), while the expression of *PP2C34* (evm.TU.Contig28.377) was higher in both NW and UW. The expression of *SNF1-related protein kinases* (*SnRK2s*) (*evm.TU.Contig16.279; evm.TU.Contig16.280*) was upregulated in both UW and LW samples (Figure 5). Upregulation of *SnRKs* in UW resulted in the expression of *ABA binding factor* (*ABF*)-associated unigenes, such as *ABF4* (*evm.TU.Contig477.648*), which finally activated ABA-dependent stress responses in *N. cadamba* stems.

Jasmonate is synthesized from lipids in the chloroplast membrane, particularly through the lipoxygenase (*LOX*) pathway to form α-linolenic acid [56]. Many important members of the *LOX* family responsible for the conversion of linolenic acid into other different polymers were differentially expressed in this study (Figure 6). For example, *LOX6* (*evm.TU.Contig154.406*) was upregulated in both UW and LW compared to NW. The activity of the *JAR1* (*evm.TU.Contig477.194*) gene, which is involved in the conversion of JA to biologically active jasmonyl-isoleucine, was high in LW (Figure 5). The main components of the JA response are transcription factors of the *JA-ZIM-domain* (*JAZ*) repressor family of genes (*TIFY4A* and *TIFY6B*, etc.), and their expression is higher in NW and LW compared to UW. The unigenes *evm.TU.Contig184.720*, *evm.TU.Contig184.722* and *evm.TU.Contig969.4* associated with *MYC2*, a transcription factor involved in the regulation of root growth and development in response to jasmonic acid stress, were highly expressed in UW and LW. Allene oxide cyclase (*AOC*) and allene oxide synthase (*AOS; evm.TU.Contig477.572*), which are involved in jasmonate synthesis, were equally expressed in all studied samples.

Many genes related to gibberellic acid (GA) synthesis were upregulated in the UW of *N. cadamba*. The GA 3 oxidase-related *evm.TU.Contig797.155* was more highly expressed in NW than in LW and UW samples (Figure 5). Two unigenes, *evm.TU.Contig51.78* and *evm.TU.Contig797.34,* encoding *Ent*-kaurenoic acid oxidase 1 (*KAO1*) involved in the conversion of *ent*-kaurenoic acid to gibberellin 12 were highly expressed in UW and LW. The novel.1016 encoding geranylgeranyl pyrophosphate synthase, an enzyme involved in the conversion of dimethylallyl diphosphate (*DMAPP*) to geranylgeranyl pyrophosphate, was highly expressed in UW, while *evm.TU.Contig14.96*, also associated with the same enzyme, had similar expression in all samples (Figure 6). The expression of GA receptors, namely *GID1b* (*evm.TU.Contig421.435*), was upregulated in UW, while the expression of *GID1c* (evm.TU.Contig244.13) was downregulated in the UW samples. The expression of *GAI1* (*evm.TU.Contig55.380*) was downregulated in UW, while *evm.TU.Contig298.41* associated with GAI was highly upregulated in both UW and LW samples (Figure 5). The expression of *SCARECROW*-like protein (*SCLs*) transcription factor associated with *DELLA* proteins was upregulated in the UW samples (Figure 5). Expression of other *DELLA* protein complexes was not detected in any of the wood samples. The downregulation/non-expression of *DELLA* proteins indicated degradation or conformational changes in *DELLA* proteins after binding of GA to the *GID1* receptor. Furthermore, the GA-related protein *GASA* (*GASA1*; *evm.TU.Contig14.234*) was found to be highly expressed in NW, but expressed weekly in LW and UW, while the expression of GASA3 (*evm.TU.Contig45*.519) was upregulated in LW samples and *GASA11* (*evm.TU.Contig66.1355*) in UW samples (Figure 5).

#### 2.7.6. Co-Expression Network

Building co-expression networks revealed a strong correlation between cell-wall biosynthesis, transcriptional factors, cellular sugar synthesis-related genes, and G layer-forming genes. *3-phosphoshikimate 1-carboxyvinyltransferase 1* (*EPSPS*-1) is a transferase that acts as a major hub gene regulating the activity of genes associated with G layer synthesis. *FLA17* is strongly correlated with the *EPSPS*-1 gene, as well as the cellulose synthesis-related gene CesA4 and the G layer synthesis-related gene *CTL2*. *BGAL* genes were highly correlated with hemicellulose synthesis-related genes such as *IRX7* and *TBL3*. The *farnesyl pyrophosphate synthase 1* (*FPS*) gene was correlated with *BGAL5* and *BGAL3*, while *FLA8* was correlated with *phospholipase D alpha 1* (*PLD1*) (Figure 6). A strong correlation was observed between cell wall synthesis-related genes and G layer-forming genes.

## 3. Discussion

Wood formation is a complex process involving multiple complex steps ranging from cambial cell division to cell function specificity, the deposition of secondary walls, and heart wood formation, all of which are controlled by internal and external factors [57,58,59]. Gravistimulation leads to the formation of reaction wood, whose formation process is significantly different from that of NW formation. The current study used RNA sequencing to investigate changes in gene expression during the early growth of *N. cadamba* bent stems in both erect/control plants and plants bent at 90° positions. In addition, microscopic visualization of the G layer and spectroscopic analysis were used to understand G layer development (Figure 1). Although gravistimulation caused severe mechanical stress, no distinct G layer was observed in the UW of *N. cadamba* (Figure 2). This is consistent with previous research on tropical rainforest angiosperm species that produce TW without a G layer [2,12].

The cell wall is considered to be a vital component of the plant response to various biotic and abiotic stresses. The composition and content of cell wall components also change during wood development, and changes in the content of these polymers determine the type of wood. For example, TW with G layers is characterized by a high cellulose content and low lignin content, and analysis of the differential expression of cellulose and lignin synthesis genes may provide a clue to understanding the mechanism of TW formation [18,60]. In the present study, no significant changes in cellulose and lignin content were observed, as revealed by FT-IR analysis, and therefore it could serve as evidence for no apparent G layer in *N. cadamba* (Figure 2). Furthermore, high expression of *CesA* promoted cellulose synthesis and the accumulation of cell wall carbohydrates in the G layer of trees, as reported in Populus having a G layer in TW fiber [61,62]. In the present study, the non-significant change in cellulose content coupled with the small change in *CesA* gene expression in UW compared to NW suggests that *N. cadamba* may contain a G layer in TW, but it may be lignified early or synthesized in very low quantities (Figure 4D,E). Lignin in the G layer is responsible for the creation of tensile strength and for the regulation of cellulose microfibril angles [63]. In contrast to the cellulose content, the G layer possesses a low lignin content [64], which may be due to the downregulation of lignin-synthesizing genes. In the present study, the slight variation in lignin content between UW and NW is in line with the expression of genes responsive to lignin synthesis. There was an increased expression of syringyl lignin-related genes in NW, such as *F5H*, *CoCOMAT*, and *COMT*, thus leading to an increase in S-lignin similar to [65] and a slight decrease in total lignin content. However, in the UW samples, as revealed by microscopic analysis, the amount of lignin was not reduced and was the same as in UW, indicating either little or no G layer content (Table S1).

Candidate proteins that are mainly responsible for G layer formation are focused on because they regulate important processes during G layer formation. For example, *FLA* and *CTL* genes regulate cellulose crystallinity and cellulose microfibril angle, respectively, during G layer formation, while the binding of these genes to *BGAL* genes also regulates gel formation [24].

The high expression of *FLA* proteins directly affects cellulose deposition and thus has an impact on the structural properties of the plant cell wall [30,66,67]. Furthermore, *FLA* genes are involved in signaling G layer formation by breaking down their *GlcNAc* oligosaccharides via the action of chitinases. G-layer formation is thought to be induced by the cleavage of *GlcNAc* oligosaccharides by the action of chitinases, as observed in flex fibers, where a higher expression of *FLAs* may also be involved in triggering a cellular signal [36,68]. The cellulose microfibril angle regulated by the G-layer is an important determinant of the mechanical and physical properties of wood, as the cellulose microfibril angle of the S2 layer regulates the tensile strength, stiffness and shrinkage of wood [69]. In the present study, the expression of many *FLA* genes was lower in UW than in NW, suggesting that the G layer may not be present or lignified in UW (Table S4).

*BGAL* genes are considered to be responsible for the modification of the RG-I pectin structure, which leads to gel formation and consequently to the tensioning of microfibrils due to gel swelling [19]. In aspen, the main large matrix retained by cellulose microfibrils was β-(1→4)-galactan and type II arabinogalactan, which were only present in TW samples. This study also reported the involvement of these *BGAL* genes in wood differentiation and the regulation of cell wall properties in TW [9]. In the present study, *BGAL* genes were downregulated in UW, while they were upregulated in NW, indicating the absence of a G layer in UW and the non-generation of tensile strength caused by the swelling of microfibrils (Figure 5A).

Plants respond to gravity by reorienting their organs; gravity is sensed by gravity-sensing cells and receptors, which process signals downstream. Amyloplasts can act as statoliths because their dense accumulation of starch granules allows them to sediment in the direction of gravity. Phosphoglucomutase (*PGM*) is involved in starch synthesis, and mutations in *PGM* in Arabidopsis lead to reduced sedimentation of amyloplasts, thereby reducing the response of shoots and roots to gravity [70]. The higher expression of *PGM* in UW samples suggests more sedimentation of amyloplasts and increased gravity response in UW compared to LW and NW samples. Similarly, *JACKDAW*, which controls the endodermis/cortex patterning and forms a ternary complex with other gravity-regulating genes (e.g., with members of *GRAS* domain transcription factors [71,72]), was expressed more in LW than in UW, thus suggesting a greater loss of quiescent center cells in UW compared to LW, resulting in the loss of tissue layers [73]. The upregulation of these *SCR*-like genes suggests that they may be involved in growth and recovery during bending stress [52]. *SHR* and *SCR* genes are key regulators of both the specification of the root stem cell niche and the differentiation potential of a subset of stem cells in the Arabidopsis root [74].

The expression of several *XTH* genes has been reported to increase in TW during their development compared to NW [8]. The increased expression of *XTH* genes in UW indicates the occurrence of high xyloglucan and the accumulation of non-cellulosic polysaccharides during gravistimulation (Table S4). High xyloglucan is related to the tight adhesion of cell wall layers and the perception and transmission of stress signals [75,76]. In the current study, *XTH* genes were less expressed in UW samples than in NW samples, in contrast to previous studies in which *XTH* activities were upregulated in the G layer and remained functional for several years [8]. This also serves as evidence that G layer formation is inhibited in *N. cadamba*.

Since wood in plants develops from the vascular cambium, the rate of wood formation is mainly regulated by cambial activity, which in turn is regulated by the activity of many plant hormones. The role of ethylene in stimulating cambial activity has been established in many studies [77]. Ethylene is essential for TW formation because it is involved in the induction of the G layer and determines the amount of G-fiber formation and its properties during TW formation [78]. Ethylene responses in plants are mainly regulated by *ERFs*; for example, in Arabidopsis, these are required for ethylene-induced cambial cell division (*ERF1, ERF018* and *ERF109*) and xylem development (*AtERF1* and AtERF2) [79,80,81]. In agreement with the literature, many ethylene biosynthesis genes, such as *EIN, ERFs, ETRs* and *ERS*, are highly expressed in UW samples of *N. cadamba* (Figure 5). Ethylene-related transcriptional factors regulate the expression of cell wall synthesis-related genes and affect wood development in a more targeted manner [82].

Gibberellin (GA) plays many important roles in the bending stress process by indirectly participating in TW formation [8,83] through the upregulation of key genes that stimulate auxin and brassinosteroid responses [84]. *DELLA* protein is a negative regulator of GA signaling and acts immediately after the GA receptor to suppress *FLA* expression under normal conditions and inhibit TW formation. There is crosstalk between ethylene and GA during stem bending; however, ethylene-responsive genes can also work independently during TW induction [85]. Brassinosteroid is also an important plant hormone involved in the regulation of TW formation and vascular development. Excess brassinosteroid levels significantly increased secondary growth and TW formation and vice versa in Populus [86]. It also negatively regulates stem bending in *Arabidopsis*, but induces the elongation of existing cells on the lower side of the stem [87,88]. In Poplar, brassinosteroid-induced stem bending is accompanied by the formation of new cells from which contractile fibers are differentiated [89]. In *N. cadamba*, all brassinosteroid-responsive genes including *BK, BIN* and *BES1/BZR* were upregulated in both UW and LW samples (Figure 5). Auxin is the most important plant hormone involved in bending stress and its subsequent distribution of auxin control stem bending in many plants. *PIN* proteins are located in the cambium and in its derived cells of Populus stem, and are responsible for polar auxin transport [90,91], whereas in *Arabidopsis*, these are expressed in the meristems of shoots and roots and maintain cell-specific auxin concentrations [92]. The auxin distribution in shoot lateral organs (e.g., leaves and branches) is maintained by the joint action of various auxin-responsive transports (e.g., *LAX* 1-3, *PIN1* and *AUX1*) [93,94]. In the present study, we detected the downregulation of auxin transports, such as *AUX22*, *PIN* genes and auxin synthesis-related genes such as *ARFs* (Figure 5). The expression of *auxin response factors* (*ARFs*) in different herbaceous and wood plants or in different organs of the same plant induces tropism and differential growth [95], whereas exogenous application of *IAA* increases the expression of cellulose synthesizing genes and TW formation by accelerating intercellular polar auxin transport. There is much evidence in the literature indicating that these hormones coordinate or that their signaling pathways coincide at one or more points to regulate cambial activity and cell division. For example, many developmental processes, such as auxin redistribution and transport during lateral root development in Arabidopsis, are regulated by cytokinin and ethylene crosstalk [96,97].

The co-expression network showed that genes related to cell wall synthesis were strongly correlated with G layer-forming genes. Similarly, genes related to cell wall extension, nucleotide sugar biosynthesis and hormonal signaling were also closely correlated with G layer-forming cells. These results suggest that cell wall-related genes, especially those involved in xyloglucan and cellulose synthesis, are important for tension generation in G layer formation [9].

## 4. Materials and Methods

### 4.1. Sample Collection and Treatment

Tissue-cultured plantlets of *N. cadamba* were grown in 15 L pots supplemented with compost and soil in a glasshouse with a 16 h day with an average temperature of 25 °C and an 8 h night with an average temperature of 18 °C. The plantlets were grown straight for 120 days, and bamboo canes were used to keep plants upright and prevent any bending. After 120 days, the pots were divided into two groups, i.e., control and horizontally bent plants. The control plants were kept straight, while the bent plants were placed horizontally at a 90° angle tied along the bamboo canes in short intervals to prevent a geotropic response in the meristems and prevent mechanical/load stress [98]. The reason for bending at 90° was that at this angle, a thicker G layer is formed, as seen in *Acacia mangium* [99]. After 30 days of bending treatment, the whole bent sections (about 5–10 cm in length of the inclined stems) were cut manually and debarked. The sliced plant piece was then cut vertically into two parts, i.e., upper side of the bent stem here referred as upper wood (UW) and lower side of the bent stem here referred as lower wood (LW). Likewise, three samples for both sides were taken from straight-standing or non-stressed trees, referred to as NW, and as no anatomical distinction was seen in NW from both sides, only one set of three samples was used for RNA-Seq analysis. All samples were immediately dipped into liquid nitrogen for DNA and RNA isolation. Another set of samples was dipped in water for microscopic observations, while for FT-IR analysis, samples were dried and ground into powder.

### 4.2. Microscopy

The wood sample preparation and staining were performed as follows [99]. Briefly, soon after cutting from the plant, the sections were fixed in formalin-acetic acid–alcohol (37% formalin–glacial acetic acid and 70% alcohol = 5 mL:5 mL:90 mL) solution for seven days. After washing with ethanol gradient, the samples were fixed on paraffin. About 10 µm-thick sections made by using a rotary microtome were later stained with Wiesner stain (phloroglucinol-HCl), which was made by dissolving 0.3 g of phloroglucinol in 10 mL of absolute ethanol to yield a 3% phloroglucinol solution. Finally, one volume of concentrated HCl was mixed with two volumes of 3% phloroglucinol in ethanol to make phloroglucinol-HCl. The stained sections were observed with an Olympus BX51 compound microscope and an Olympus DP74 digital camera for imaging. The images were further processed by using CELLSENS software (Olympus Corp., Tokyo, Japan).

### 4.3. Fourier Transform Infrared Spectroscopy (FT-IR)

The FT-IR spectra of a debarked stem section that was powdered, pressed, and ground into a tablet containing 1 mg of the wood sample and 100 mg of potassium bromide (KBr) were recorded on a spectrophotometer in the range of 2000–600 cm^−1^. The samples were kept dry, and data were recorded in three replicates and finally averaged. The method used to build the infrared spectra was previously described by Zhu et al. [100].

### 4.4. RNA Extraction, Sequencing and Statistical Analysis

The stem sections from bent as well as control plants were collected and stored at −80 °C. The stem tissues were ground using motorized tissue grinders and RNA isolation was performed using the RNAprep Pure Assay kit (Tiangen, Beijing, China) following the standard manual. The quantity and purity (1.8 < OD260/280 < 2.2) of the samples were checked using a Nanodrop spectrophotometer to confirm the suitability of the samples for library preparation. The integrity of RNA was checked using an RNA Nano 6000 Assay Kit in the Agilent 2100 Bioanalyzer System, and samples with an RNA integrity number (RIN) ≥7.5, an RNA concentration ≥300 ng/μL, and a total RNA amount ≥2 μg were used for further sequencing. Sequence libraries were created using the NEBNext^®^ UltraTM RNA Library Prep Kit from Illumina^®^ (NEB, Ipswich, MA, USA) following the guidelines from the manufacturers. The detailed methods are described in our previous reports [40,101]. The samples were sent to Novogene technology Co., Ltd. (Beijing, China) for RNA-Seq analysis. The sequences from each wood sample were cleaned using the FASTX toolkit with a minimum quality threshold of 20 and unknown nucleotides >5%. Shorter reads were first combined with longer reads based on their paired-end information similarity between contigs before being mapped back to the contigs using the Trinity platform. The longest chain was used as the sample unigene, and sequences of all unigenes were submitted to the Transcriptome Shotgun Assembly Sequence Database (TSA) with the *NCBI* submission number *CRA004124* (https://bigd.big.ac.cn/gsub/, accessed on: May 2021). Finally, the transcripts with fpkm values less than 5 were filtered out and the remaining transcripts were used for data analysis. The *EdgeR* package was used to perform differential gene expression, and significance *p* values (≤0.05) were checked using the Benjamini and Hochberg method. For the creation of heatmaps, the *Complexheatmap* package in *R* software was used. 

### 4.5. Enrichment Analysis

The functional enrichment analysis including GO analysis was performed to identify DEGs significantly associated with each GO category or term. *ClusterProfiler* in *R* was used to perform GO enrichment analysis; GO terms with FDR ≤ 0.05 were considered significantly enriched. The KEGG pathway analysis was executed to retrieve the enriched pathways with *p*-value ≤ 0.05.

### 4.6. Co-Expression Network

A correlation analysis in *R* was used to identify genes and TFs that were strongly correlated with genes associated with G layer synthesis. Initially, the highly expressed genes (fpkm > 20) were filtered out of the entire dataset, and correlation analysis was performed. Only highly correlated genes (r^2^ > 0.97 and *p* value ≤ 0.005) from the results were imported into *cytoscape* software to create a co-expression network.

## 5. Conclusions

The bending stress caused serious alterations in the transcriptome profile of *N*. *cadamba* seedlings. Gravistimulation caused the downregulation of many genes and transcriptional factors involved in cell wall and hormone biosynthesis. Induction of a G layer was inhibited by the downregulation of *FLA* and *BGAL* genes. These genes were highly correlated with cell-wall-synthesis-related genes and some sugar substrates responsible for the synthesis of cell wall polymers. The increased flux of sugar, ions and amino acid transportation was due to rapid asymmetric growth towards the upper side of the stem. Finally, the downregulation of various cambium synthesizing genes was also recorded. Altogether, the present study provides detailed insights into the mechanism of G layer inhibition in *N*. *cadamba*, and further biochemical and molecular studies are required to validate these results.

## Data Availability

The data presented in this study are available on request from the corresponding authors.

## Supplementary Materials

The following supporting information can be downloaded at: https://www.mdpi.com/article/10.3390/ijms24010268/s1.
