# Supplementary material for: Transcriptomic Evidence Reveals Low Gelatinous Layer Biosynthesis in Neolamarckia cadamba after Gravistimulation
Source: Int J Mol Sci. 2022 Dec 23;24(1):268. doi: 10.3390/ijms24010268 (PMC9820806; doi:10.3390/ijms24010268)
Supplement: Supplementary file 1 [file ijms-24-00268-s001.zip › ijms-2017559-supplementary.pdf]

Table S1: Relative content (%age) of cell wall polymers in all studied samples

Cell wall polymers	Lower wood (LW)	Upper wood (UW)	Normal wood (NW)
Lignin	21.79	23.50	23.46
Hemicellulose	29.59	27.47	26.77
Cellulose	48.62	49.03	49.77

Table S2 Quality evaluation of RNA seq Data

Sample	Raw reads	Clean reads	Clean bases	Error rate	Q20	Q30	GC pct
NW1	49523950	48448364	7.27G	0.03	97.48	92.99	43.45
NW2	44078816	43027212	6.45G	0.03	97.74	93.61	43.53
NW3	47313792	46323534	6.95G	0.03	97.67	93.41	43.36
LW1	52040510	51020920	7.65G	0.03	97.85	93.78	43.52
LW2	47437012	46470654	6.97G	0.02	98.04	94.27	43.56
LW3	45535078	44791128	6.72G	0.03	97.72	93.53	43.17
UW1	45997564	45103602	6.77G	0.03	97.71	93.56	42.92
UW2	47152244	46344026	6.95G	0.03	97.94	93.98	43.47
UW3	45644496	44509336	6.68G	0.02	98.07	94.21	42.8

Table S3 Alinement and statistics of clean reads on reference genome.

sample	total reads	total map	unique map	multimap	read1_map	read2_map
NW1	48448364	45924153(94.79%)	44548981(91.95%)	1375172(2.84%)	22392500(46.22%)	22156481(45.73%)
NW2	43027212	40924035(95.11%)	39706375(92.28%)	1217660(2.83%)	19899410(46.25%)	19806965(46.03%)
NW3	46323534	43912692(94.8%)	42575422(91.91%)	1337270(2.89%)	21363902(46.12%)	21211520(45.79%)
LW1	51020920	48266752(94.6%)	46752195(91.63%)	1514557(2.97%)	23454238(45.97%)	23297957(45.66%)
LW2	46470654	44280034(95.29%)	42779203(92.06%)	1500831(3.23%)	21432578(46.12%)	21346625(45.94%)
LW3	44791128	42445769(94.76%)	41155243(91.88%)	1290526(2.88%)	20643469(46.09%)	20511774(45.79%)
UW1	45103602	42427217(94.07%)	41049869(91.01%)	1377348(3.05%)	20573626(45.61%)	20476243(45.4%)
UW2	46344026	43822340(94.56%)	42391499(91.47%)	1430841(3.09%)	21268967(45.89%)	21122532(45.58%)
UW3	44509336	42132712(94.66%)	40758836(91.57%)	1373876(3.09%)	20427809(45.9%)	20331027(45.68%)

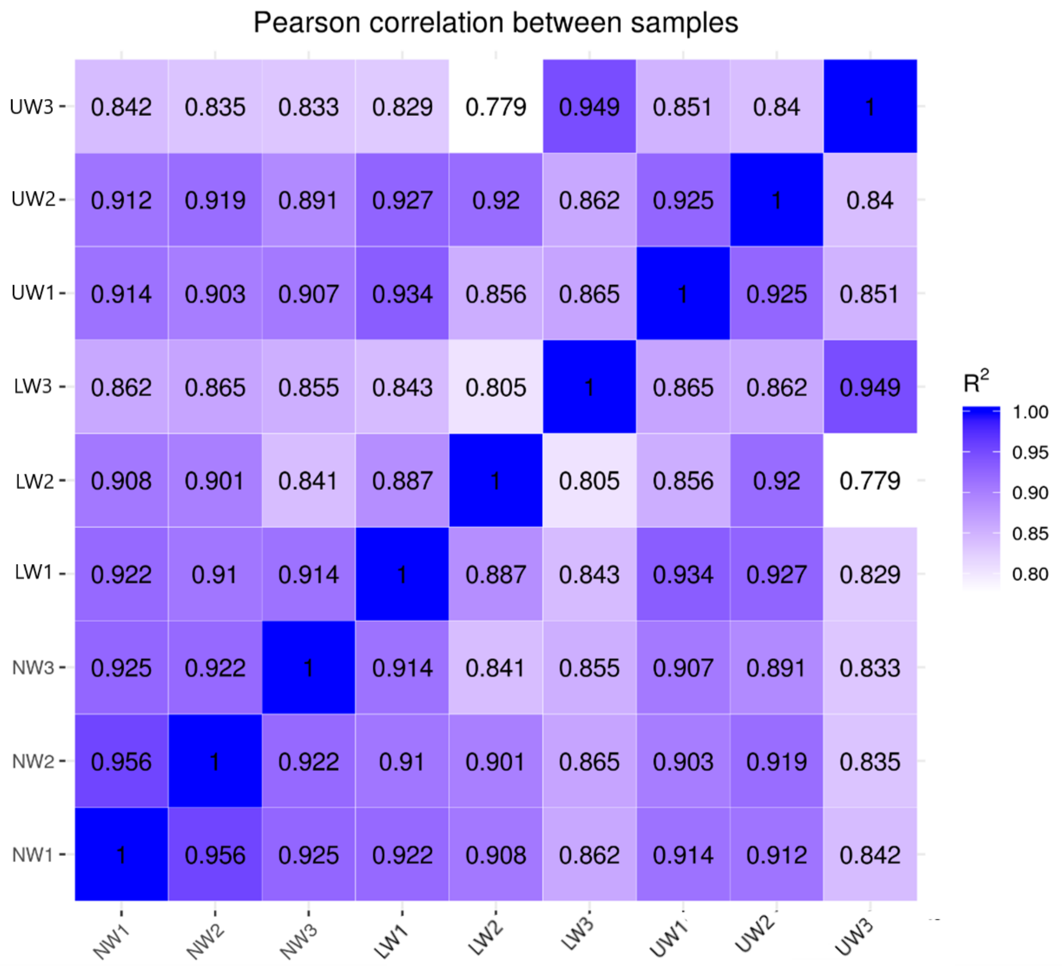

Figure S1: Pearson correlation coefficient among all three biological replicates from each samples

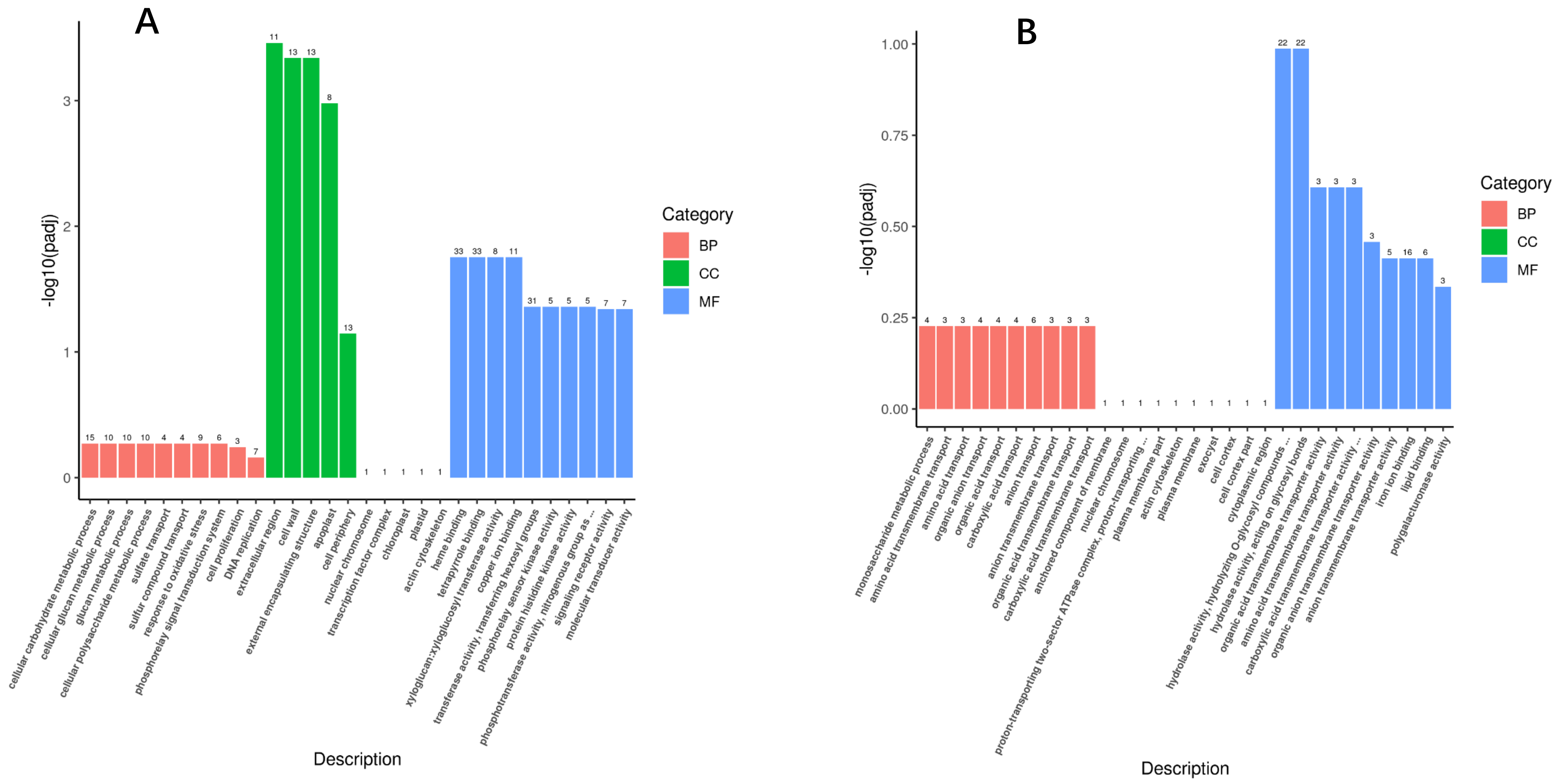

Figure S2: Go terms LWvsNW. A) Go terms for upregulated genes B) Go terms for downregulated genes

BP=Biological process CC=Cellular component and MF=Molecular function

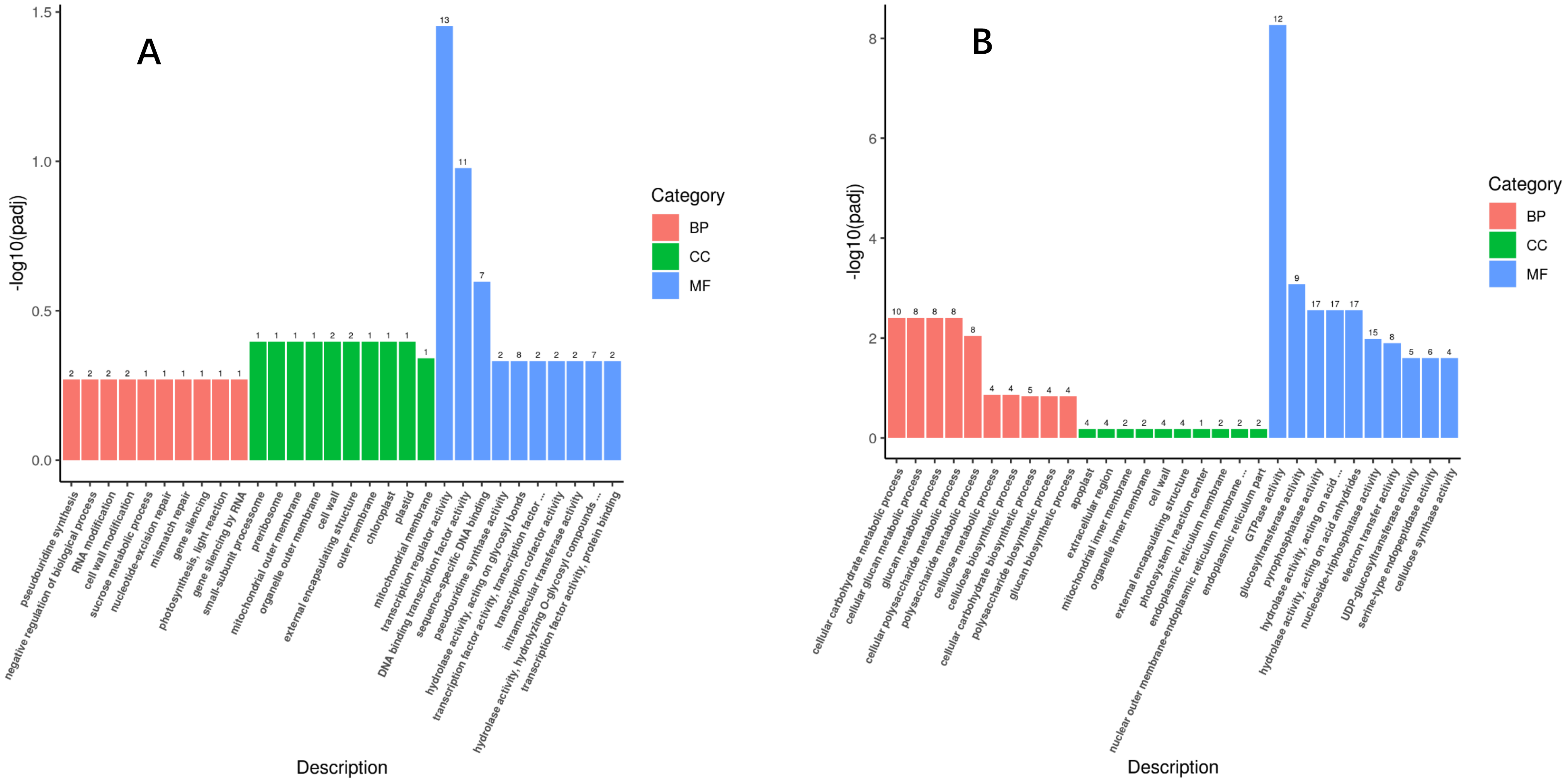

Figure S3: Go terms UWvsLW. A) Go terms for upregulated genes B) Go terms for downregulated genes

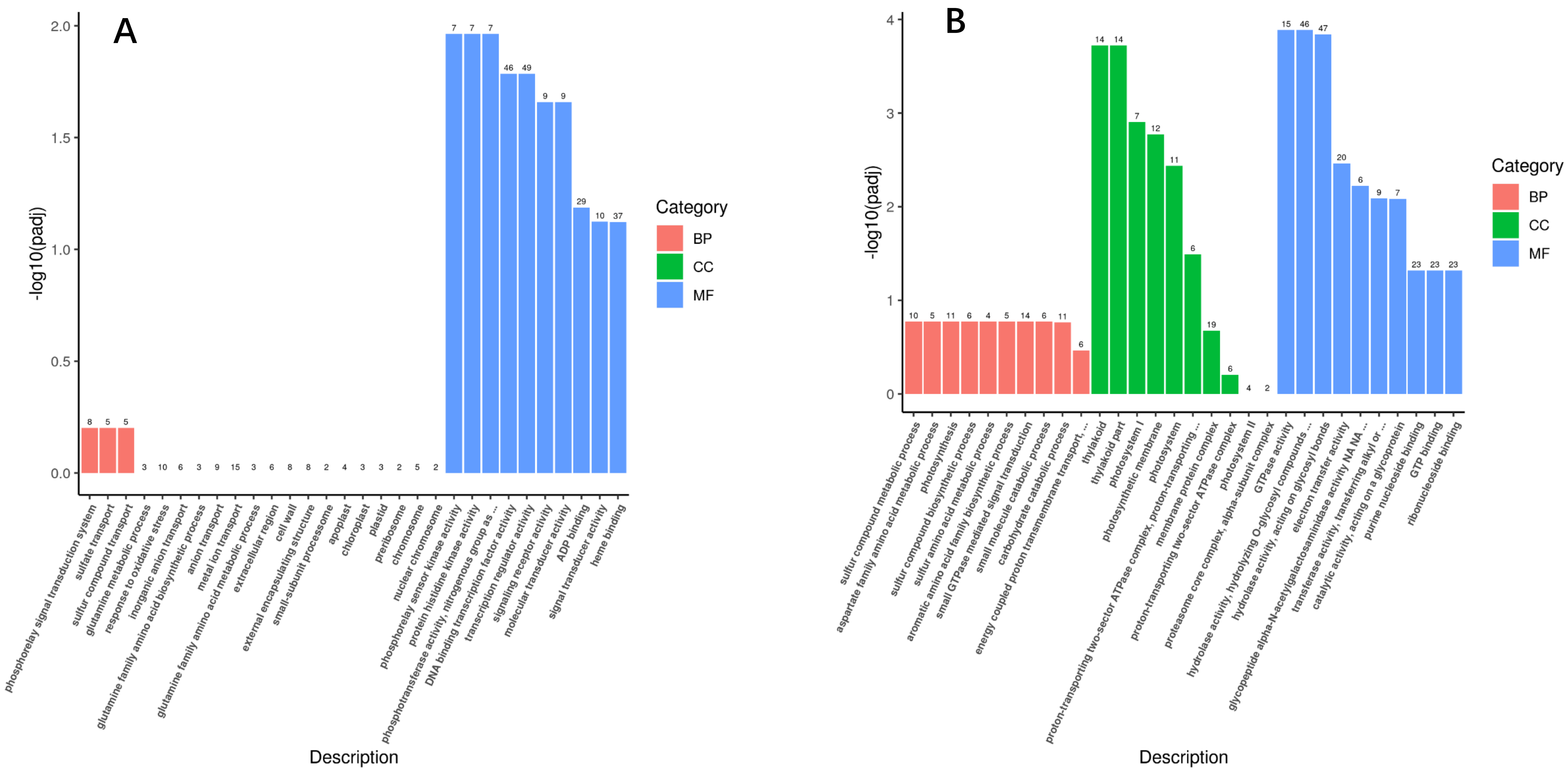

Figure S4: Go terms UWvsNW. A) Go terms for upregulated genes B) Go terms for down regulated genes

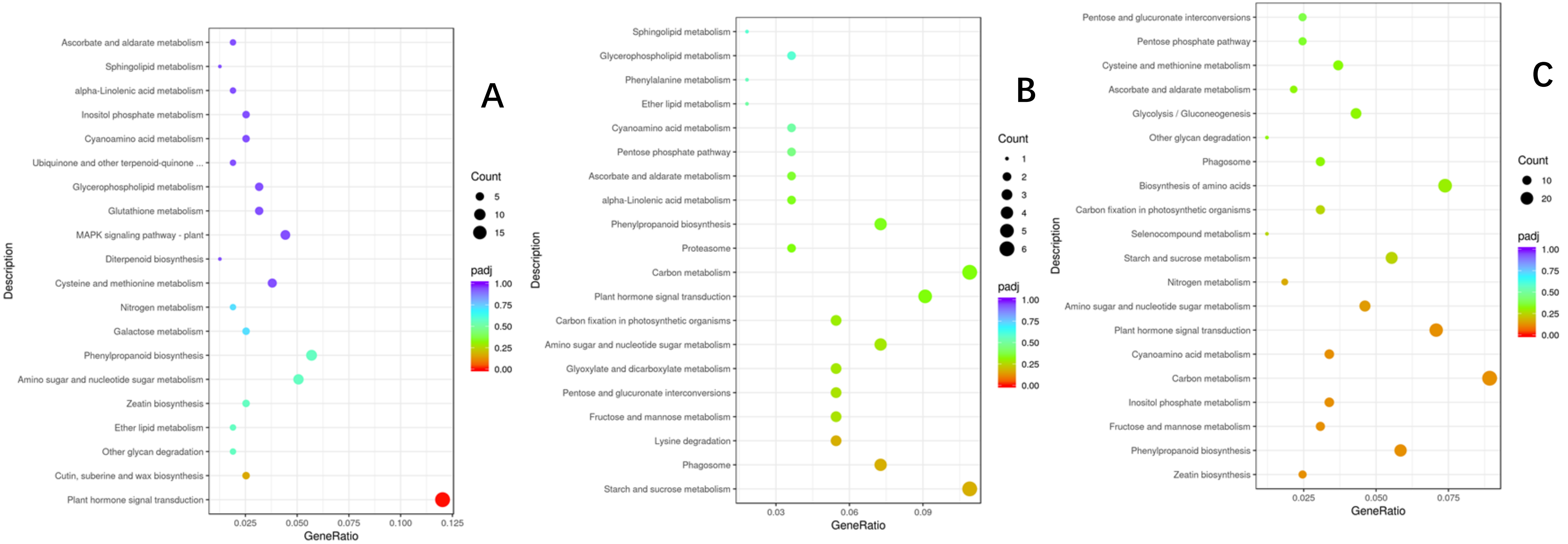

Figurer S5: KEGG pathway enrichment analysis a) LWvsNW b) UWvsLW c) UWvsNW

Table S4: Expression of gene related to sugar synthesis, transport and metabolism during bending stress in *N. cadamba*

N.cadamba gene_id	NW1	NW2	NW3	LW1	LW2	LW3	UW1	UW2	UW3	Associated gene
**evm.TU.Contig96.44**	19.08	10.14	25.94	31.06	25.20	46.43	41.97	18.10	35.28	Thermospermine synthase
**evm.TU.Contig421.510**	27.25	50.96	72.86	39.74	14.35	64.77	111.01	79.38	71.19	Beta-glucosidase 1
**evm.TU.Contig341.557**	6.73	8.53	9.35	8.57	5.42	8.26	14.43	13.81	20.35	Beta-glucosidase 11
**evm.TU.Contig421.509**	16.11	12.76	15.68	24.40	35.84	21.71	26.78	27.37	23.45	Beta-glucosidase 11
**evm.TU.Contig421.511**	18.76	39.28	53.86	35.59	9.83	35.88	68.07	56.76	51.30	Beta-glucosidase 11
**evm.TU.Contig66.81**	62.13	51.50	54.82	47.61	58.10	38.76	45.55	45.98	39.62	Beta-glucosidase 12
**evm.TU.Contig184.901**	114.13	79.08	88.94	404.67	90.64	194.79	216.18	183.28	275.67	Beta-glucosidase 17
**evm.TU.Contig81.360**	163.86	155.16	138.82	104.54	134.69	203.77	96.34	119.86	131.61	Chorismate synthase 1, chloroplastic
**evm.TU.Contig256.101**	304.37	413.25	300.87	265.52	264.82	297.48	217.66	254.60	182.62	3-phosphoshikimate 1-carboxyvinyltransferase
**evm.TU.Contig797.212**	85.70	83.19	71.86	63.50	70.95	75.56	58.74	62.83	56.70	P23981.1 3-phosphoshikimate 1-carboxyvinyltransferase 1
**evm.TU.Contig14.86**	188.79	174.62	151.67	111.02	115.70	150.98	115.01	83.96	86.45	Probable galacturonosyltransferase-like 1
**novel.906**	249.14	257.63	220.90	155.75	217.35	263.07	171.00	173.23	188.58	Probable galacturonosyltransferase 12
**evm.TU.Contig421.306**	20.36	24.16	27.35	18.19	17.53	45.09	42.97	27.17	40.72	Probable galacturonosyltransferase 6
**evm.TU.Contig3.73**	218.47	226.21	210.02	95.41	101.08	169.32	96.70	62.25	133.40	UDP-glucuronatexylan alpha-glucuronosyltransferase 1
**evm.TU.Contig555.387**	127.24	165.92	127.64	128.74	98.02	118.93	122.71	70.47	86.35	UDP-glucuronatexylan alpha-glucuronosyltransferase 2
**evm.TU.Contig16.459**	156.21	147.90	124.66	110.77	95.91	115.58	100.40	68.95	76.55	Probable polygalacturonase
**evm.TU.Contig477.134**	51.80	66.49	87.05	23.98	17.70	47.08	18.55	16.06	37.87	Probable polygalacturonase
**evm.TU.Contig600.201**	71.30	78.69	61.16	58.78	45.65	84.93	54.62	38.70	45.06	Probable polygalacturonase
**evm.TU.Contig22.204**	7.77	9.62	8.33	10.47	13.37	12.27	15.65	13.86	14.56	UDP-glucose iridoid glucosyltransferase

N.cadamba gene_id	NW1	NW2	NW3	LW1	LW2	LW3	UW1	UW2	UW3	Associated gene
**evm.TU.Contig477.772**	32.54	24.96	20.14	20.44	32.93	7.50	12.21	23.08	12.21	Probable xyloglucan endotransglucosylase/hydrolase protein 10
**evm.TU.Contig63.448**	69.68	31.26	42.70	36.65	66.12	13.65	18.44	27.36	11.83	Probable xyloglucan endotransglucosylase/hydrolase protein 32
**evm.TU.Contig138.26**	99.74	114.20	77.93	133.98	236.46	179.85	99.79	148.06	113.20	Xyloglucan endotransglucosylase/hydrolase protein 9
**evm.TU.Contig12.412**	78.13	134.28	144.50	38.13	37.32	70.29	26.64	23.63	56.05	Anti-sigma-I factor RsgI6
**evm.TU.Contig131.49**	31.49	29.67	29.53	22.43	29.67	26.37	20.12	20.94	20.78	Phosphoserine phosphatase
**evm.TU.Contig14.221**	356.96	306.10	232.70	217.38	238.55	334.66	175.03	179.91	204.26	Endoglucanase 10
**evm.TU.Contig16.723**	19.58	25.95	69.96	22.01	12.21	17.54	14.37	12.42	19.91	Inactive beta-amylase 9
**evm.TU.Contig16.766**	24.53	29.50	22.51	26.27	27.29	184.07	39.05	28.90	150.47	Glutamine synthetase leaf isozyme, chloroplastic
**evm.TU.Contig184.275**	26.39	27.22	31.43	28.48	36.67	16.42	21.52	21.50	12.82	Spermidine synthase
**evm.TU.Contig184.626**	5.53	9.88	13.78	13.52	10.78	22.77	17.74	15.00	27.37	UDP-glycosyltransferase 73E1
**evm.TU.Contig21.419**	419.31	395.45	418.18	353.72	441.57	364.80	308.65	328.10	348.26	Glutamine synthetase nodule isozyme
**evm.TU.Contig214.15**	77.87	66.04	65.57	54.72	85.90	47.23	48.27	56.66	33.40	Serine hydroxymethyltransferase 4
**evm.TU.Contig245.66**	39.23	32.42	20.06	40.08	42.96	37.80	53.91	57.88	59.70	Beta-hexosaminidase 3
**evm.TU.Contig33.24**	10.23	11.31	6.09	25.30	63.64	13.32	24.77	20.28	13.24	Beta-galactosidase 10
**evm.TU.Contig33.52**	59.16	61.04	54.60	52.84	63.58	44.26	40.12	49.26	32.09	Fructose-bisphosphate aldolase 3, chloroplastic
**evm.TU.Contig331.24**	53.50	44.82	28.33	33.63	84.21	16.32	13.36	34.66	18.42	Endoglucanase 8
**evm.TU.Contig341.556**	14.76	14.42	19.18	15.72	9.06	13.95	23.64	25.99	42.95	Cyanidin 3-O-glucoside 5-O-glucosyltransferase (acyl-glucose)
**evm.TU.Contig341.628**	200.50	180.48	142.89	178.52	364.42	54.07	148.43	93.90	53.54	Probable beta-D-xylosidase 2
**evm.TU.Contig387.25**	886.65	570.40	602.37	505.27	600.95	688.63	231.31	350.00	324.86	Serine hydroxymethyltransferase 4
**evm.TU.Contig387.26**	1661.42	1530.26	1395.51	902.05	1153.84	1611.05	639.64	737.83	958.06	Serine hydroxymethyltransferase 4
**evm.TU.Contig39.23_evm.TU.Contig39.24**	50.37	42.10	41.87	98.86	264.26	21.56	58.07	61.86	17.76	Lysosomal beta glucosidase

N.cadamba gene_id	NW1	NW2	NW3	LW1	LW2	LW3	UW1	UW2	UW3	Associated gene
**evm.TU.Contig394.291**	58.76	58.41	40.93	50.83	141.04	20.73	22.09	46.98	28.83	Beta-galactosidase 3
**evm.TU.Contig401.2**	15.85	17.04	13.30	20.58	27.42	26.88	22.17	28.92	34.03	Beta-hexosaminidase 3
**evm.TU.Contig447.325**	62.16	64.77	57.94	52.11	60.67	54.30	43.57	52.29	43.06	Pyruvate kinase isozyme A
**evm.TU.Contig462.308**	148.62	220.12	249.38	66.23	58.36	155.00	44.62	34.72	99.85	Mannan endo-1,4-beta-mannosidase 6
**evm.TU.Contig471.211**	10.58	10.33	11.87	14.77	26.38	12.16	11.65	12.12	13.83	Plastidial pyruvate kinase 2
**evm.TU.Contig471.249**	49.67	43.62	41.81	37.88	38.29	41.55	35.01	30.07	34.99	Spermine synthase
**evm.TU.Contig477.309**	19.03	18.89	19.14	21.16	22.43	18.51	26.28	23.28	23.57	Probable galacturonosyltransferase 3
**evm.TU.Contig477.391**	46.24	41.12	29.93	20.91	18.27	22.07	31.41	32.10	42.10	Probable endo-1,4-beta-xylanase C
**evm.TU.Contig480.237**	83.89	58.48	75.50	88.16	251.73	68.15	52.19	69.79	57.86	Probable glucan endo-1,3-beta-glucosidase A6
**evm.TU.Contig481.85**	96.12	107.45	89.11	117.57	169.72	181.15	123.32	156.85	117.83	Fructose-bisphosphate aldolase 3
**evm.TU.Contig555.158**	29.67	28.32	26.92	26.99	35.95	21.03	23.26	20.50	20.97	Hexokinase-2
**evm.TU.Contig585.15**	716.17	593.11	521.76	562.16	734.40	365.20	363.25	416.42	317.12	Fructokinase-2
**evm.TU.Contig63.482**	26.94	27.79	24.64	25.13	29.15	18.35	20.66	19.57	18.92	Isocitrate dehydrogenase [NAD] catalytic subunit 5, mitochondrial
**evm.TU.Contig66.167**	6.06	7.33	5.99	7.14	8.63	9.99	8.00	9.65	9.00	Isocitrate dehydrogenase [NADP], chloroplastic/mitochondrial
**evm.TU.Contig66.950**	94.29	95.85	80.35	78.21	49.88	51.75	68.22	28.56	37.66	Serine hydroxymethyltransferase, mitochondrial
**evm.TU.Contig69.10**	33.09	27.10	47.80	31.42	27.19	10.18	22.37	15.65	11.08	Beta-amylase 1
**evm.TU.Contig69.9**	23.70	19.47	28.30	22.55	19.09	8.03	11.66	9.29	8.03	Beta-amylase 1
**evm.TU.Contig7.272**	420.50	424.99	347.56	401.67	598.54	478.86	300.32	391.25	314.98	Fructose-bisphosphate aldolase 6
**evm.TU.Contig906.31**	158.40	184.51	124.81	113.46	204.42	124.29	76.05	121.51	52.73	Fructose-bisphosphate aldolase 6
**evm.TU.Contig985.20**	112.63	142.13	145.06	50.69	57.24	81.66	43.95	26.92	80.73	Beta-L-arabinofuranosidase
**novel.32**	221.78	251.07	356.82	212.09	76.90	180.12	213.96	141.33	101.89	UDP-glucoronosyl and UDP-glucosyl transferase
**novel.36**	12.83	10.96	7.11	14.11	24.38	14.69	12.48	26.50	26.23	UDP-glycosyltransferase 74E2

Table S5: Expression of cell wall esterases and signalling molecules expressed during bending stress in *N. cadamba*

Gene id	NW1	NW2	NW3	LW1	LW2	LW3	UW1	UW2	UW3	Gene name	GH Family	Gene description
**evm.TU.Contig138.26**	99.74	114.20	77.93	133.98	236.46	179.85	99.79	148.06	113.20	XTH9	GH16	Xyloglucan endotransglucosylase/hydrolase protein 9
**evm.TU.Contig14.119**	5.86	7.41	5.17	11.22	18.74	8.84	8.57	12.57	8.97	NA	GH17	Glucan endo-1,3-beta-glucosidase 7
**evm.TU.Contig14.221**	356.96	306.10	232.70	217.38	238.55	334.66	175.03	179.91	204.26	At1g75680	GH9	Endoglucanase 10
**evm.TU.Contig16.459**	156.21	147.90	124.66	110.77	95.91	115.58	100.40	68.95	76.55	NA	GH28	Probable polygalacturonase
**evm.TU.Contig16.723**	19.58	25.95	69.96	22.01	12.21	17.54	14.37	12.42	19.91	BAM1	GH14	Inactive beta-amylase 9
**evm.TU.Contig184.901**	114.13	79.08	88.94	404.67	90.64	194.79	216.18	183.28	275.67	AA5GT	GH1	Beta-glucosidase 17
**evm.TU.Contig207.323**	27.42	25.66	8.33	37.09	51.52	36.14	18.18	43.28	29.61	LAC2	Laccase	Laccase-2
**evm.TU.Contig245.66**	39.23	32.42	20.06	40.08	42.96	37.80	53.91	57.88	59.70	HEXO3	GH20	Beta-hexosaminidase 3
**evm.TU.Contig278.41**	50.01	95.59	79.05	43.15	33.68	38.72	41.21	41.22	26.33	BGAL9	GH35	Beta-galactosidase 9
**evm.TU.Contig296.267**	110.00	116.91	66.23	139.91	169.02	141.50	94.04	193.19	90.00	LAC2	Laccase	Laccase-2
**evm.TU.Contig296.367**	78.79	226.99	154.59	121.32	555.98	352.42	110.06	509.78	138.95	LAC17	Laccase	Laccase-17
**evm.TU.Contig33.24**	10.23	11.31	6.09	25.30	63.64	13.32	24.77	20.28	13.24	BGAL10	GH35	Beta-galactosidase 10
**evm.TU.Contig331.24**	53.50	44.82	28.33	33.63	84.21	16.32	13.36	34.66	18.42	CEL1	GH9	Endoglucanase 8

**evm.TU.Contig341.556**	14.76	14.42	19.18	15.72	9.06	13.95	23.64	25.99	42.95	AA5GT	GH1	Cyanidin 3-O-glucoside 5-O-glucosyltransferase (acyl-glucose)
**evm.TU.Contig341.557**	6.73	8.53	9.35	8.57	5.42	8.26	14.43	13.81	20.35	BGLU11	GH1	Beta-glucosidase 11
**evm.TU.Contig394.291**	58.76	58.41	40.93	50.83	141.04	20.73	22.09	46.98	28.83	BGAL3	GH35	Beta-galactosidase 3
**evm.TU.Contig401.2**	15.85	17.04	13.30	20.58	27.42	26.88	22.17	28.92	34.03	HEXO3	GH20	Beta-hexosaminidase 3
**evm.TU.Contig421.509**	16.11	12.76	15.68	24.40	35.84	21.71	26.78	27.37	23.45	BGLU11	GH1	Beta-glucosidase 11
**evm.TU.Contig421.510**	27.25	50.96	72.86	39.74	14.35	64.77	111.01	79.38	71.19	BGLU1	GH1	Beta-glucosidase 1
**evm.TU.Contig421.511**	18.76	39.28	53.86	35.59	9.83	35.88	68.07	56.76	51.30	BGLU11	GH1	Beta-glucosidase 11
**evm.TU.Contig462.160**	491.41	378.37	382.31	361.90	370.84	368.85	352.37	266.51	299.55	LAC10	Laccase	Laccase-10
**evm.TU.Contig462.308**	148.62	220.12	249.38	66.23	58.36	155.00	44.62	34.72	99.85	MAN6	GH5	Mannan endo-1,4-beta-mannosidase 6
**evm.TU.Contig462.71**	238.29	130.22	124.27	140.89	196.41	97.88	117.58	79.71	59.58	LAC17	Laccase	Laccase-17
**evm.TU.Contig464.29**	14.03	15.43	9.21	12.83	16.49	70.68	13.47	18.33	97.86	At5g56590	GH28	Aspartic proteinase-like protein 1
**evm.TU.Contig477.134**	51.80	66.49	87.05	23.98	17.70	47.08	18.55	16.06	37.87	NA	GH10	Probable polygalacturonase
**evm.TU.Contig477.391**	46.24	41.12	29.93	20.91	18.27	22.07	31.41	32.10	42.10	xlnC	GH16	A1CHQ0.1 Probable endo-1,4-beta-xylanase C
**evm.TU.Contig477.772**	32.54	24.96	20.14	20.44	32.93	7.50	12.21	23.08	12.21	XTH10	GH17	Probable xyloglucan endotransglucosylase/hydrolase protein 10
**evm.TU.Contig480.236**	191.19	120.65	184.74	171.85	509.68	124.67	117.98	128.80	81.50	At1g11820	GH17	Glucan endo-1,3-beta-glucosidase 1
**evm.TU.Contig480.237**	83.89	58.48	75.50	88.16	251.73	68.15	52.19	69.79	57.86	A6	GH28	Probable glucan endo-1,3-beta-glucosidase A6
**evm.TU.Contig600.201**	71.30	78.69	61.16	58.78	45.65	84.93	54.62	38.70	45.06	NA	GH16	Probable polygalacturonase
**evm.TU.Contig63.448**	69.68	31.26	42.70	36.65	66.12	13.65	18.44	27.36	11.83	XTH32	GH1	Probable xyloglucan endotransglucosylase/hydrolase protein 32
**evm.TU.Contig66.1552**	39.89	38.47	35.72	34.23	32.10	29.61	25.43	22.49	19.52	CXE11	GH14	Probable carboxylesterase 11
**evm.TU.Contig66.1553**	33.43	32.76	25.92	26.32	26.66	27.46	20.50	19.71	19.85	CXE11	GH14	Probable carboxylesterase 11

**evm.TU.Contig66.81**	62.13	51.50	54.82	47.61	58.10	38.76	45.55	45.98	39.62	BGLU12	Laccase	Beta-glucosidase 12
**evm.TU.Contig69.10**	33.09	27.10	47.80	31.42	27.19	10.18	22.37	15.65	11.08	NA	GH127	Beta-amylase 1; chloroplastic
**evm.TU.Contig69.9**	23.70	19.47	28.30	22.55	19.09	8.03	11.66	9.29	8.03	BAM1		Beta-amylase 1; chloroplastic
**evm.TU.Contig96.209**	258.15	394.02	307.81	252.84	209.50	280.12	220.29	173.91	186.54	LAC22		Laccase-22
**evm.TU.Contig985.20**	112.63	142.13	145.06	50.69	57.24	81.66	43.95	26.92	80.73	NA	GH127	Beta-L-arabinofuranosidase,
